# The genome assembly of the fungal pathogen *Pyrenochaeta lycopersici* from Single-Molecule Real-Time sequencing sheds new light on its biological complexity

**DOI:** 10.1371/journal.pone.0200217

**Published:** 2018-07-06

**Authors:** Alessandra Dal Molin, Andrea Minio, Francesca Griggio, Massimo Delledonne, Alessandro Infantino, Maria Aragona

**Affiliations:** 1 Dipartimento di Biotecnologie, Università degli Studi di Verona, Verona, Italy; 2 Consiglio per la ricerca in agricoltura e l’analisi dell’economia agraria, Research Centre for Plant Protection and Certification, Rome, Italy; Ruhr-Universitat Bochum, GERMANY

## Abstract

The first draft genome sequencing of the non-model fungal pathogen *Pyrenochaeta lycopersici* showed an expansion of gene families associated with heterokaryon incompatibility and lacking of mating-type genes, providing insights into the genetic basis of this “imperfect” fungus which lost the ability to produce the sexual stage. However, due to the Illumina short-read technology, the draft genome was too fragmented to allow a comprehensive characterization of the genome, especially of the repetitive sequence fraction. In this work, the sequencing of another *P*. *lycopersici* isolate using long-read Single Molecule Real-Time sequencing technology was performed with the aim of obtaining a gapless genome. Indeed, a gapless genome assembly of 62.7 Mb was obtained, with a fraction of repetitive sequences representing 30% of the total bases. The gene content of the two *P*. *lycopersici* isolates was very similar, and the large difference in genome size (about 8 Mb) might be attributable to the high fraction of repetitive sequences detected for the new sequenced isolate. The role of repetitive elements, including transposable elements, in modulating virulence effectors is well established in fungal plant pathogens. Moreover, transposable elements are of fundamental importance in creating and re-modelling genes, especially in imperfect fungi. Their abundance in *P*. *lycopersici*, together with the large expansion of heterokaryon incompatibility genes in both sequenced isolates, suggest the presence of possible mechanisms alternative to gene re-assorting mediated by sexual recombination. A quite large fraction (~9%) of repetitive elements in *P*. *lycopersici*, has no homology with known classes, strengthening this hypothesis. The availability of a gapless genome of *P*. *lycopersici* allowed the in-depth analysis of its genome content, by annotating functional genes and TEs. This goal will be an important resource for shedding light on the evolution of the reproductive and pathogenic behaviour of this soilborne pathogen and the onset of a possible speciation within this species.

## Introduction

*Pyrenochaeta lycopersici* is a hemibiotrophic fungus belonging to the large class of *Dothideomycetes*. It is pathogenic to tomato and other agronomically important Solanaceous species [1,2]. The pathogen is the agent of Corky Root Rot (CRR), a disease widespread especially under intensive tomato production systems and in greenhouse, with yield losses of 30–40% or more [3,4]. The pathogen attacks the main root causing the typical corky aspect, but the disease, especially in the field, tends to be underestimated, due to the lack of significant symptoms on the aerial parts of the plant. *P*. *lycopersici* has a soilborne behaviour and it can persist in the soil for several years by producing vegetative resting structure, the sclerotia. Previous analyses have revealed the existence of two different biotypes of *P*. *lycopersici* (i.e. Type 1 and Type 2) on the basis of growth morphology and rate in culture, ribosomal DNA internal transcribed spacer (rDNA-ITS) and diagnostic specific primers [5,6], random amplified polymorphic DNA (RAPD) [7] and amplified fragment length polymorphism (AFLP)-based population analysis [8]. Despite the same shape and size of conidiophores and conidia shared by the two *P*. *lycopersici* biotypes, low similarity (89–90%) of ITS sequences, and genetic variation among populations have been observed. Considering these evolutionary features between the two biotypes, new insights into *P*. *lycopersici* biology are needed. Recently, Type 2 *P*. *lycopersici* ER1211 isolate was sequenced using paired-end Illumina technology [9]. This allowed to obtain useful information on the genic protein-coding regions of this non-model pathogen and to report the first observations on the expansion of some gene families relevant for the biology of this fungus. However, the short-read-based strategy did not permit to represent in the final assembly the highly fragmented fraction of *P*. *lycopersici* genome, including also the repetitive sequences. Indeed, the correct assembly of repetitive elements is a difficult task in large genomes mainly because of their length. Based on their mechanisms of transposition, TEs are ordered in two main classes, which are themselves split into orders and several super families, families, and sub-families [10]. Class I elements (e.g., LTRs, DIRS and LINE) transpose via RNA intermediates while Class II elements (e.g., TIR, Crypton and Helitron) transpose directly from DNA. Other categories considered in this classification include non-autonomous TEs, like LARD, TRIM and MITEs.

In the last years, several authors demonstrated the link of non-coding DNA to traits controlling the life behaviour and evolution of fungi [11,12], and the interest in these regions is increasing. As a consequence, the complete assembly of a genome is of fundamental importance to the field of genome structure and evolution of these organisms [13,14]. Repetitive regions as transposable elements, in addition to the role of specific genes, are also involved in regulation of fungal pathogenicity [15,16]. Thus, great advantages are emerging from the ability of third generation sequencing technologies in resolving long repeats, especially in the study of non-model organisms with no available reference genome. The availability of a well-assembled genome of *P*. *lycopersici* is also an important pre-requisite for discovering new putative effectors, usefulness for the improvement of control measures to CRR disease, both in the field and greenhouse. At present, after the ban of soil fumigation with methyl bromide and of other ozone-depleting substances, the common systems to control CRR include soil solarisation and grafting on disease resistant rootstock, but they can be used only in the greenhouse and their effect is limited in presence of high levels of inoculum in the soil. Source of partial resistance to CRR was identified in wild tomato species [17,18], but has rarely been introgressed into commercial tomato varieties.

In this study, the Type 1 ER1518 *P*. *lycopersici* isolate was sequenced using PacBio RS long-read technology. A gapless genome assembly was obtained and analysis of the genomic data allowed to: i) formulate new considerations about the expansion of some protein families potentially involved in pathogenicity and reproduction of this species; ii) perform a comparative analysis of annotated transposable elements with other fungi of the same *phylum*; iii) discover some putative effector-like molecules and a transcription factor having effector features in other pathogens of Pleosporales order [19,20].

## Materials and methods

### Sample preparation and SMRT cells sequencing

Genomic DNA was isolated from a virulent *P*. *lycopersici* isolate (CRA-PAV_ER 1518) according to Cenis [21] and modified as reported by Aragona *et al*. [9]. Genomic DNA was quantified with Qubit dsDNA HS Assay kit (Life Technologies), purity and integrity of DNA were assessed with Nanodrop 1000 spectrophotometer (Thermo Scientific) and by agarose gel electrophoresis. The extracted DNA was approximately 20 kb long and, sticking to the criteria requested for pure DNA, was directly used for SMRTbell^™^ libraries creation at Keygene (Wageningen, the Netherlands). Eight SMRT Cells were generated and sequenced by the PacBio RS II system using P5-C3 chemistry and a 180-minute data collection mode.

### Genome assembly

Assembly of the long genomic reads was performed using HGAPv3 software [22] on a local implementation of SMRTportal (ver. 2.2). Library pre-filtering was performed with standard parameters (*Minimum Subread Length* = 500bp, *Minimum Polymerase Read Quality* = 0.80, *Minimum Polymerase Read Length* = 100bp), while multiple sets of assembly parameters where tested in order to reduce the fragmentation of the assembly. The best assembly, in terms of number of contigs in front of longest assembled sequences, was obtained using the *Minimum Seed Read Length* = 3000bp, *Target Coverage* = 15, *Number Of Seed Read Chunks* = 6, *Alignment Candidates Per Chunk* = 10, *Overlapper K-Mer* = 14 and the other parameters were left at default values. Polishing of the assembly was performed using Quiver with uniquely mapping reads only.

Alignment of Type 2 ER1211 *P*. *lycopersici* genome assembly [9] was performed with MUMMER software [23] while genomic reads were aligned using BWA (ver. 0.7.10-r789) with *mem* algorithm and default parameters.

### Gene prediction and annotation

The *ab initio* prediction of protein-coding gene sequences was performed with Genemark ES ver. 4.10 [24], using the masked genome sequence for training and setting a minimum contig length of 200. Functional annotation of predicted protein-coding gene sequences was performed with BLAST ver. 2.2.28+ [25] against the NCBI Non-Redundant (NR) database retrieved on 2015-02-02 (E-value <1e-06) and the Uniprot SwissProt Fungi protein database retrieved on 2014-05-29 (E-value <1e-07). The sequences were functionally annotated using Blast2GO (ver. 2.8) [26] with default parameters. Gene models were further annotated for conserved protein domains by using HMMer ver. 3.1b [27] and Pfam database (ver. 26.0, retrieved 2011-11-01). Hits in Pfam database were considered significant at an e-value threshold <1e-06 for both the entire sequence match and for the independent E-value of the single domain match. Gene models were annotated for putative homology to carbohydrate-active enzymes (CAZymes) in dbCAN (ver. 3.0) database using HMMer ver. 3.1b. Alignments were considered significant with an alignment length > 80 residues, E-value < 1e-05 and HMM profile coverage > 30% or alignment length < 80 residues and E-value <1e-03 and HMM profile coverage > 30%. BLAST ver. 2.2.28+ [25] was used to identify putative homologies to known pathogenic genes (PHIbase ver. 3.2), peptidases (MEROPS ver. 9.8), Mating-Type sequences [28] and membrane transport proteins (Transporter Classification Database, ver. 2011-July-15) using an E-value cut-off <1e-10. All predicted protein sequences were analysed using Phobius ver. 1.01 [29] to predict if they were likely to be signal peptides and then with EffectorP ver. 2.0 [30] to test if they were predicted effectors.

### Orthologous genes analysis

Orthologous groups were determined using OrthoMCL software (ver. 2.0.9) [31]. A protein database of 137,752 predicted protein sequences from *P*. *lycopersici* CRA-PAV_ER1518, *P*. *lycopersici* CRA-PAV_ER1211, *Aspergillus nidulans* ASM1142 v1.24, *Blumeria graminis* EF 1.24, *Colletotrichum higginsianum* GCA_000313795 v2.24, *Fusarium oxysporum* FO 2.24, *Leptosphaeria maculans* ASM23037 v1.24, *Neurospora crassa* ASM18292 v1.24, *Phaeosphaeria nodorum* ASM14691 v1.24, *Pyrenophora teres* GCA_000166005 v1.24, *Pyrenophora tritici repentis* GCA_000149985 v1.24 was created, based on sequences downloaded from Ensemble Fungi database (http://fungi.ensembl.org/). To determine which proteins were conserved in all species, an all-versus-all analysis was performed using BLASTP [25] using “seg” filter and an E-value threshold of 1e-05, as suggested in the guidelines of the tool. The results were processed by OrthoMCL (mcl-14-137) using default inflation factor of 1.5 and default 50% similarity cut-off.

### Phylogenetic analysis

Phylogenetic analysis of the eleven selected fungal species was performed based on the protein sequences of three single copy genes that are shared among all analysed species. Single copy clustering proteins were obtained by selecting orthoMCL groups with exactly one representative from each genome. Orthologous amino acid sequences were aligned separately using MAFFT ver. 7.402 [32] with 1000 iterative refinements. After that, columns with gaps were removed using Gblocks ver. 0.91b [33]. The evolutionary history was inferred using the Maximum Likelihood method PhyML ver. 3.0 [34] with 100 bootstrap default substitution model. The visualization of the phylogenetic tree has been done with MEGA7 [35].

### Repeat annotation and masking

REPET pipeline ver. 2.2 [36] was used to detect TE sequences in the *P*. *lycopersici* ER1518 genome. A consensus sequence for each TE family was provided and classified by REPET TEdenovo according to particular features, such as structural features or homology with known TE, HMM profiles or host genes. For this homology search, the nucleotide and amino acid sequences of characterized TEs of Repbase database (ver. 18.08) and the HMM profile bank coming from Pfam ver. 26.0 (retrievable from the REPET website) were used. TEdenovo tool uses PASTEClassifier [37] to classify the repeat consensus sequences according to Wicker's classification [10]. After that, REPET TEannot tool annotated TE genomic copies using the previously obtained TE consensus library. The genomic sequence was masked with bedtools ver. 2.17.0 [38] using the final repeat library. The AT-rich regions of *P*. *lycopersici* ER1518 genome assembly were evaluated using the software tool OcculterCut ver. 1.1 [39]. OcculterCut starts by segmenting the assembled genome into regions of differing GC-content using the Jensen–Shannon divergence (DJS). Then, the segments are categorized as either AT-rich or GC-equilibrated respect to a cut-off GC value set as the local minimum between the two peaks in a Cauchy distribution mixture model [39].

## Results and discussion

In this work, we present the gapless genome of the tomato root pathogen *P*. *lycopersici* ER1518, sequenced using 3^rd^ generation technology. The availability of a gapless genome allowed to predict and annotate not only the protein coding gene sequences, but additionally the transposable elements.

### *De novo* genome sequencing and assembly

In total, 8 SMRT cells were used for *P*. *lycopersici* ER1518 DNA sequencing, yielding a total of 6.67 Gb in 1,202,336 reads with a mean length of 5.6 Kb, an N50 length of 12.6 Kb and a median coverage of 69x. The assembly produced 188 gapless unitigs covering a total genome sequence of 62.7 Mb. Assembly statistics are reported in Table 1.

**Table 1:** Statistics of *P*. *lycopersici* ER1518 genome assembly.

Assembly length (bp)	62,731,747
Number of unitigs	188
GC content (%)	46
Average sequence length (Kbp)	333.7
Minimum sequence length (Kbp)	10.4
Maximum sequence length (Kbp)	2,540
N50 (Kbp)	1,076.4
N90 (Kbp)	214.4

Mapping of filtered PacBio reads on the polished assembly aligned 94.7% of the dataset for a total of 4.87 Gb and a mean read depth of 75.8x. CEGMA analysis [40] showed that the genome assembly represented 238 complete ultra-conserved core eukaryotic genes (CEGs) out of 248 (96%), increasing to 242 when considering at least a partial match (S1 Table). This result was in agreement with that obtained for the previously sequenced *P*. *lycopersici* isolate [9] and helped to assess the comprehensiveness of the CEG space covered by the new sequenced genome. Comparison between the two *P*. *lycopersici* isolates showed not only that the genome size was increased by 8 Mb (12.8%) but also that the sequence fragmentation was reduced by one order of magnitude (N50 length increased from 74 Kb to 1.1 Mb), probably by virtue of SMRT-based sequencing in resolving longer repeats (S1 Fig and S2 Table). However, while the two *P*. *lycopersici* genomes showed to align for more than 97% of their length, they exhibit low sequence identity (mean 87.5%, estimated from aligned regions) (S2 Fig). This data are confirmed as well by the low percentage of Illumina raw genomic reads of ER1211 isolate mapping (~58%).

### Gene prediction and functional annotation

The prediction of *P*. *lycopersici* ER1518 genes has been performed using an *ab initio* approach, due to the unavailability of RNA-seq data. Genemark ES *ab initio* protein-coding gene prediction allowed us to identify 14,186 genes with a mean length of 1,473.55 bp and a mean number of exons of 2.78. The main structural features of *P*. *lycopersici* ER1518 gene predictions have been compared with *P*. *lycopersici* ER1211 and other nine *Ascomycetes* (Table 2) to evaluate the “goodness” of the obtained *in silico* annotation. The gene prediction of *P*. *lycopersici* ER1211 has been performed using the same software version of ER1518 isolate in order to make the gene annotations comparable. Average transcript length, median intergenic distance and mean intron length are nearly identical between the two *P*. *lycopersici* isolates. Even if the number of genes in *P*. *lycopersici* ER1518 is slightly higher respect to ER1211 isolate, *P*. *lycopersici* ER1518 has less monoexonic genes, probably indicating a better resolution at sequence level in the new assembled genome.

**Table 2:** Statistics of *P*. *lycopersici* ER1518 gene prediction.

	*PLY* *ER1518*	*PLY* *ER1211*	*AN*	*BG*	*CH*	*FO*	*NC*	*PN*	*LM*	*PTT*	*PTR*
***Number of genes***	14,186	14,058	5,032	6,470	16,141	17,696	9,820	12,372	12,469	11,799	12,169
***Mean intergenic distance (bp)***	289.91	-75.93	-301.12	4,923.65	11.99	425.59	81.43	-78.89	93.81	-268.50	-200.26
***Median intergenic distance (bp)***	832	801	693	5,363.50	681.50	1061	1,269	728	638	681	758
***Mean gene length (bp)***	1,473.55	1,434.01	1,536.64	1,587.31	1,092.85	1,345.05	1,529.48	1,316.24	1,255.48	1,387.96	1,423.31
***Mean number of exons***	2.78	2.63	3.40	2.77	2.44	2.70	2.74	2.67	2.82	2.50	2.67
***Mean Exon length (bp)***	530.81	544.71	451.92	573.77	447.03	497.44	557.36	493.83	444.72	554.27	533.59
***Median Exon length (bp)***	315	335	240	337	257	287	266	294	213	311	305
***Mean intron length (bp)***	95.02	100.30	88.79	67.27	85.72	100.48	134.89	89.13	102.05	88.62	113.40
***Max intron length (bp)***	4,399	8,348	1,286	1,468	2,564	2,369	1,273	1,107	5,774	5,049	2,107
***Number of monoexonic genes***	3,542	3,989	639	1,332	5,111	4,392	1,832	2,263	3,016	3,176	2,726
***Percentage of monoexonic genes (%)***	25	28	12	21	32	25	19	29	24	27	22

Comparison of gene structural features of *Pyrenochaeta lycopersici* ER1518 (PLY ER1518) with other 10 ascomycetes. Abbreviations: *Pyrenochaeta lycopersici* ER1211 (PLY ER1211), *Aspergillus nidulans* (AN), *Blumeria graminis* (BG), *Colletotrichum higgisinianum* (CH), *Fusarium oxysporum* (FO), *Neurospora crassa* (NC), *Leptosphaeria maculans* (LM), *Phaeospheria nodorum* (PN, *Pyrenophora teres* (PTT) and *Pyrenophora tritici-repentis* (PTR).

*P*. *lycopersici* ER1518 mean gene length is slightly higher but in agreement with the other *Dothideomycetes*, i.e. *L*. *maculans*, *P*. *tritici-repentis* and *P*. *teres*, and also, on average, with other *Ascomycetes*, except for *B*. *graminis* that exhibits the highest value (Table 2). In addition, exon- and intron-specific features are comparable to those of other fungi. Notably, the mean intergenic distance is higher respect to all the other fungi, especially the other *Dothideomycetes*, compared to the total number of genes. This may probably indicate a more sparse distribution of *P*. *lycopersici* ER1518 genes throughout the genome and possibly the lack of identification of some genes or genes portions, maybe due to the lack of support of a RNA-seq dataset.

As further support of the gene predictions, we obtained that 13,559 genes, 95.6% of the total gene count, were functionally annotated (S3 Table). Many of these genes (91.9%) were conserved in other species, as shown by hits against sequences in the NCBI-NR protein database (e-value < 1E-06) and the SwissProt Fungi protein database (e-value < 1E-07). Based on the BLAST hits, at least one Gene Ontology term was assigned to 4,357 gene sequences (S3 Table).

**Fig 1:**
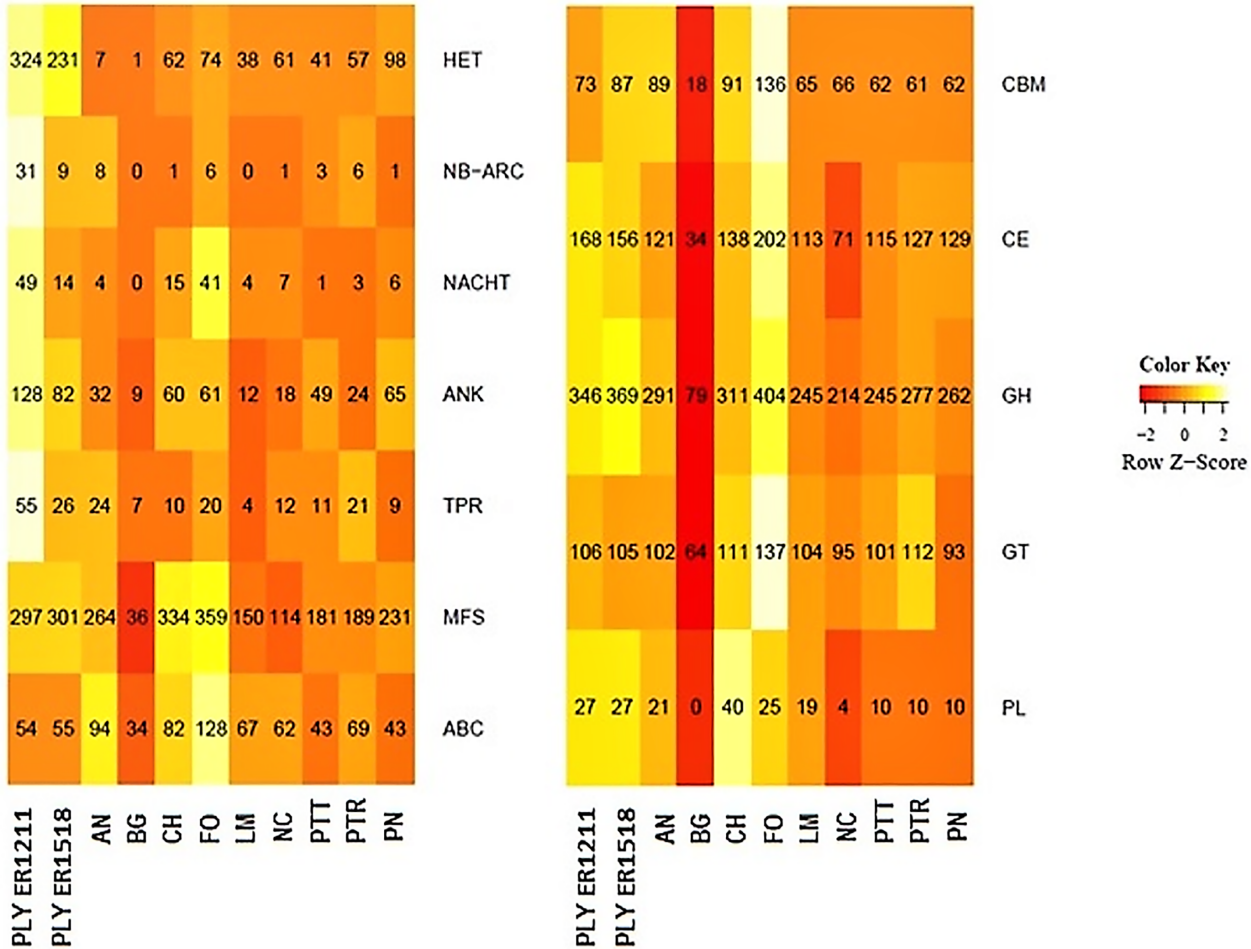
Heatmap of OrthoMCL orthologous groups for the most interesting Pfam protein and CAZymes domains identified in *P*. *lycopersici* ER1518 (PLY ER1518) and ten other fungal pathogens. The heatmap represents the type and the number of domains (rows) for each fungus (columns). The Z-score indicates that the values have been centred and scaled by rows (domains), so that negative z-scores are more likely coloured in red and high z-scores in white. Abbreviations: PLY ER1211, *P*. *lycopersici* ER1211; AN, *Aspergillus nidulans*; BG, *Blumeria graminis*; CH, *Colletotrichum higgisinianum*; FO, *Fusarium oxysporum*; LM, *Leptosphaeria maculans*; NC, *Neurospora crassa*; PTT, *Pyrenophora teres*; PTR, *Pyrenophora tritici-repentis*; PN, *Phaeospheria nodorum*. CMB, Carbohydrate-Binding Modules; CE, carbohydrate esterases; GH, Glycoside Hydrolases; GT, Glycosyl-Transferases; PL, Polysaccharide Lyases; HET, HETerokaryon Incompatibility-related domains, NB-ARC, Nucleotide-Binding Adaptor shared by APAF-1, R proteins, and CED-4 domain; NACHT, Neuronal Apoptosis inhibitor; ANK, ankyrin; TPR, tetratricopeptide; ABC, ATP-Binding Cassette transporters; MFS, Major Facilitator domains.

Among protein domains, the heterokaryon incompatibility (HET) modules are much more expanded in *P*. *lycopersici* (231 and 324 modules in ER1518 and ER 1211 isolates, respectively) compared to other fungi (Fig 1, S4 Table). An expansion is observed also for NB-ARC, NACHT, ANK and TPR domains which are functionally associated with HET and involved in Programmed Cell Death (PCD) and immune response, in agreement with that previously reported [9]. *Het* genes have been associated to the high level of variability of filamentous fungi in which vegetative reproduction predominates on sexual one [41]. In most pathogenic fungi about 50–100 HET modules mediate vegetative incompatibility between two genetically incompatible individuals so, the very large expansion in *P*. *lycopersici* of this family, suggests the importance in finding mechanisms for increasing genotypic diversity in this fungus that historically was not known to undergo sexual reproduction. In terms of ABC transporters, Major Facilitator (MFS) and CAZyme domains, *P*. *lycopersici* is more similar to other fungi of the same class, though an expansion of glycoside hydrolase (GH) and polysaccharide lyase (PL) families is noteworthy, to probably underline the importance of these components in *P*. *lycopersici* pathogenicity and virulence (Fig 1 and S5–S7 Tables).

### Orthologous genes analysis

The orthologous genes analysis, based on similarity among predicted protein sequences, identified genes shared between the two *P*. *lycopersici* isolates and also with other 10 ascomycetes, resulting in 16,307 orthologous groups (S8 Table). Functional annotation of PFAM and CAZymes protein families and domains was performed on all OrthoMCL groups resulting in a functional assignment for 10,382 (63.67%) groups.

Among all groups, 6,510 contained *P*. *lycopersici* ER1518 proteins and 172 of them included exclusively *P*. *lycopersici* ER1518 proteins, probably having similar structure and diverging from a common ancestral gene (commonly defined by OrthoMCL developers as “paralogous groups”).

Other 3,535 orthologous groups were shared with the *P*. *lycopersici* ER1211 isolate. Finally, 6,828 (48.13%) *P*. *lycopersici* proteins were not included in any orthologous group and thus referred to as “singletons” (Table 3). We checked whether these singletons had similarity with sequences annotated in public databases and if they could represent unique genetic material, putative “private” genes, conferring specific functions relevant to the ecological niche of this fungus. Among 6,828 singletons proteins, 5,974 could be assigned to a putative function and were related to KEGG pathways of biosynthesis of antibiotics and primary metabolism (S9 Table). We did not find similarity in public databases for the remaining 854 genes.

**Table 3:** Statistics of orthologous analysis performed with OrthoMCL.

Total number of orthologous groups with ten Ascomycetes	16,307
Number of groups with *P*. *lycopersici* ER1518 proteins	6,510
Number of *P*. *lycopersici* ER1518 paralogous groups	172
Number of groups in common with *P*. *lycopersici* ER1211	3,535
Number of *P*. *lycopersici* ER1518 singletons	6,828

### Phylogenetic relationships

**Fig 2:**
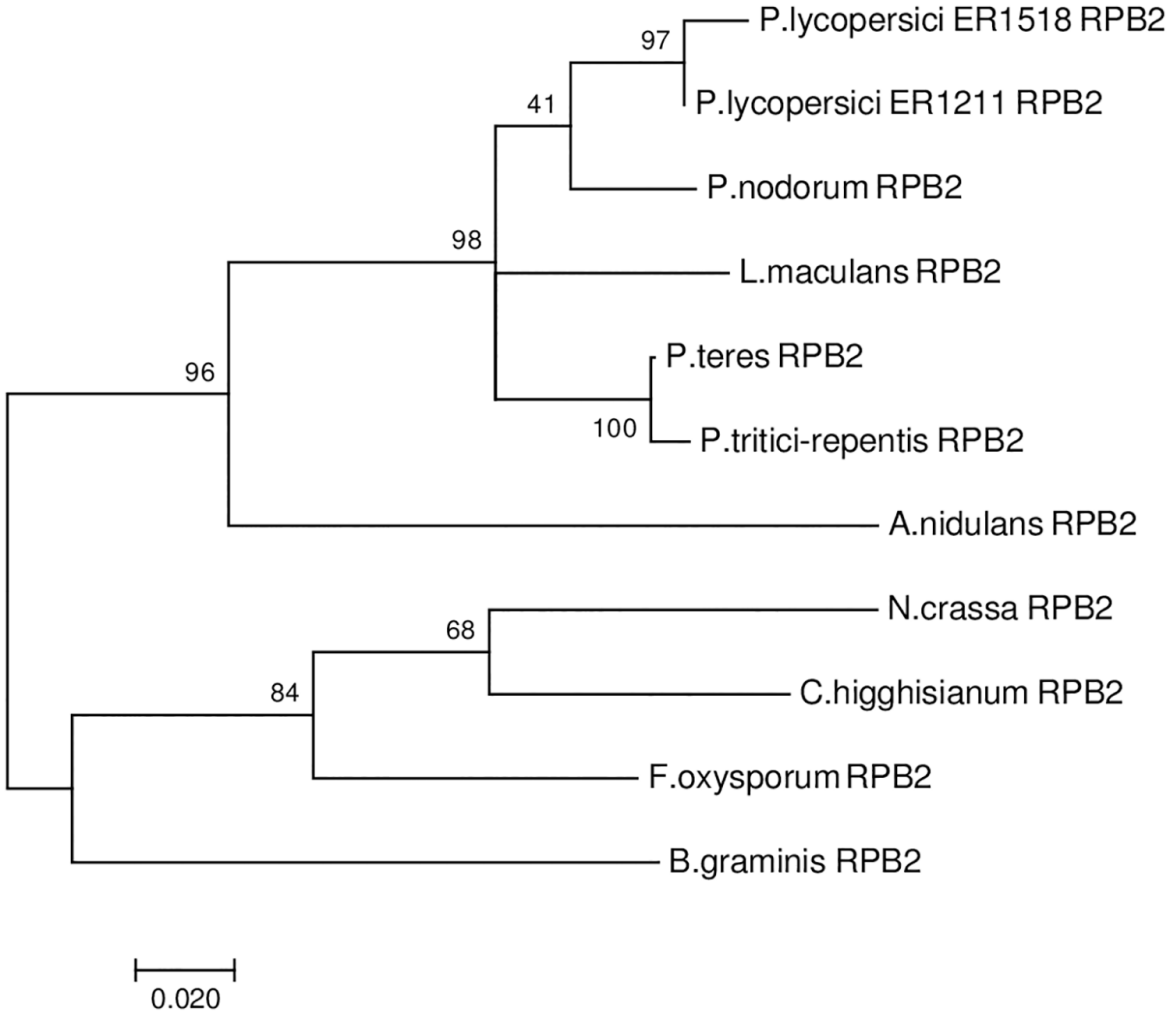
Phylogenetic tree of RPB2 protein of *P*. *lycopersici* ER1518 and other ten ascomycetes obtained with PhyML 3.0 and drawn with MEGA7. The tree is drawn to scale, with bootstrap values on branches and branch lengths measured in the number of substitutions per site.

The phylogenetic analysis was conducted on orthoMCL orthologous proteins of eleven species belonging to the four major Ascomycota classes: *Leotiomycetes*, *Dothideomycetes*, *Sordariomycetes* and *Eurotiomycetes*, generated from comparative analysis. Inside these classes, the focus was on plant pathogenic species and those with completely assembled and annotated genome sequences, which were also taken in account in previous analyses [9]. The evolutionary analysis of RPB2 gene clustered *P*. *lycopersici* ER1518 together with *P*. *lycopersici* ER1211, in the class of *Dothideomycetes* (Fig 2), and more closely related to hemibiotrophic and necrotrophic plant pathogens of the genera Leptosphaeria and Pyrenophora than to biotrophs such as the genus Blumeria, as already reported in Aragona *et al*. [9].

The close phylogenetic relationship between *P*. *lycopersici* ER1518 and *P*. *lycopersici* ER1211 and the evolutionary relationship with the other fungi classes analysed were also confirmed by the phylogenetic trees based on other two orthologous genes obtained from orthoMCL analysis (S3 and S4 Figs). These genes, coding for a AA9 (formerly GH61) and a HET protein, were chosen related to the lifestyle of this fungal pathogen and because they belong to families largely expanded in *P*. *lycopersici* genome, as discussed in next section.

### Characterization of transposable elements (TEs)

In addition to the protein coding genes sequences, a significant portion of the fungal genomes is occupied by repetitive elements [42]. Therefore, the identification and annotation of repeats has become an indispensable part of the analyses in fungal genomes sequencing projects. Recently, Amselem et al. [43] conducted a comparative analysis of transposable elements in 10 fungal genomes with different TE content, identifying species-specific associated signatures. In the present study, we performed the repeat identification and annotation on *P*. *lycopersici* ER1518 genome and other five fungal genomes using REPET *de novo* repeat identification pipeline and compared the results obtained.

**Fig 3:**
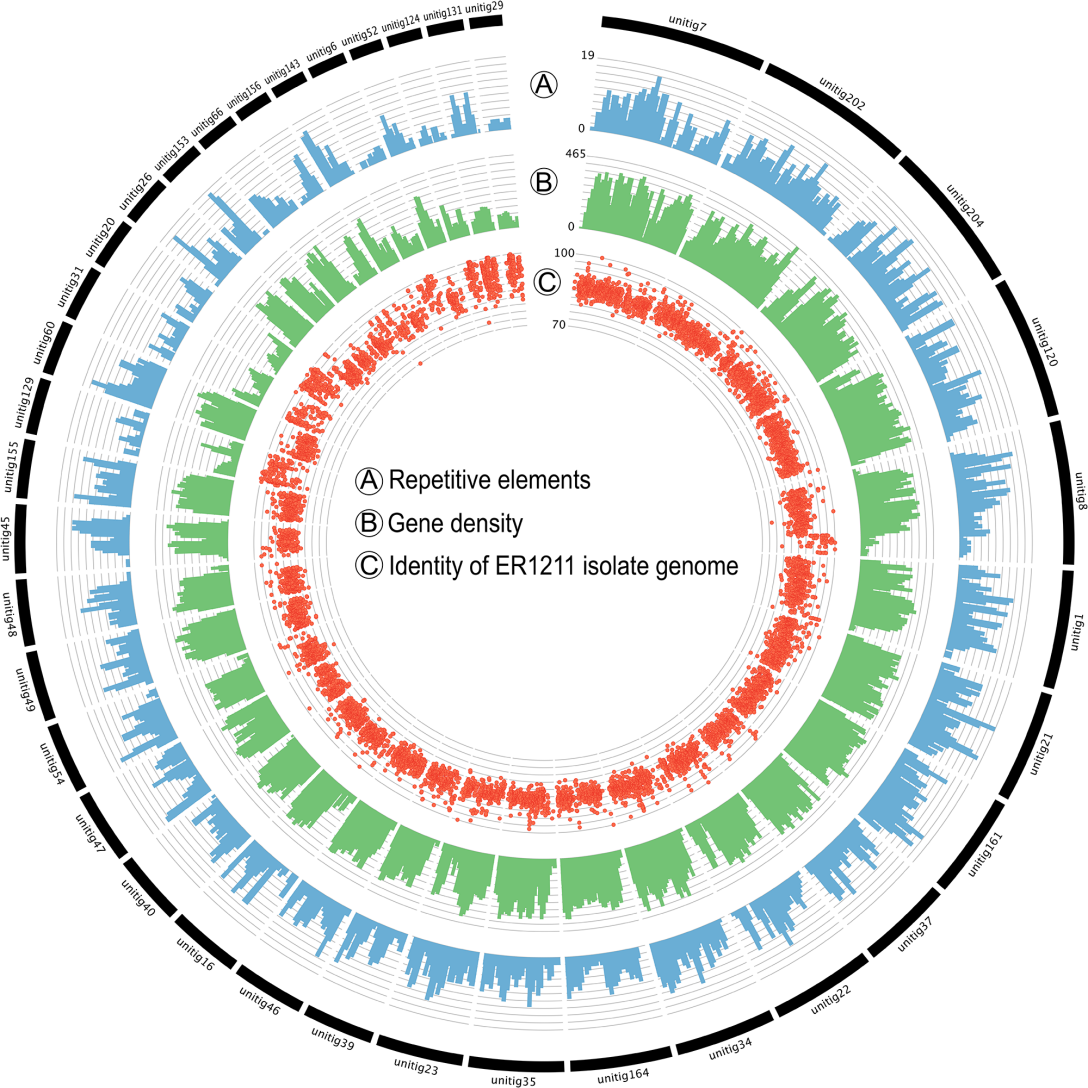
Circular representation of genomic features. Circular representation of the assembled sequences (length > 10Kb) of *P*. *lycopersici* ER1518 genome reporting the distribution of the following features: A) Repetitive elements count (blue); B) Gene density (green); C) Sequence identity percentage (red) of *P*. *lycopersici* ER1211 genomic sequences based on pairwise alignment between genome assemblies performed with MUMmer.

The annotation of repetitive sequences performed with REPET identified more than 19 Mb (30.6% of genomic sequence) of repetitive sequences throughout the genomic assembly (Fig 3 and Table 4). This percentage is slightly lower than the value reported for *L*. *maculans* and, in general, is significantly higher respect to the other four fungi (S10 Table).

**Table 4:** Statistics of repeat annotation and masking of *P*. *lycopersici*. TE classes have been reported according to Wicker classification [10].

Class	Superfamily	Genome Coverage (bp)	Average length (bp)	Number of copies (complete)
***Class I (retrotransposons)***	**LTR Copia**	1,761,880	5,999.7	122 (35)
	**LTR Gypsy**	5,850,079	7,465.3	275 (58)
	**DIRS**	160,301	8,039.3	55 (4)
	**PLE Penelope**	235,349	11,579.0	4 (0)
	**LINE**	690,466	8,733.4	28 (10)
	**SINE**	480	480.0	1 (0)
	**LARD**	1,498,181	6,046.0	59 (0)
	**TRIM**	392,991	1,453.2	33 (0)
***Class II (DNA transposons)***	**TIR Tc1-Mariner**	3,366,772	3,664.3	135 (47)
	**TIR hAT**	2,316,380	4,024.8	92 (33)
	**TIR**	606,580	3,193.3	34 (4)
	**Helitron**	914,995	9,827.4	23 (2)
	**Crypton**	79,701	4,401.0	1 (1)
	**Maverick**	64,754	14,614.5	2 (0)
	**MITE**	89,568	1,073.0	19 (0)
***Uncharacterized***		1,148,396	2,770.4	89 (0)
***TOTAL***		19,176,873	-	972 (194)

**Fig 4:**
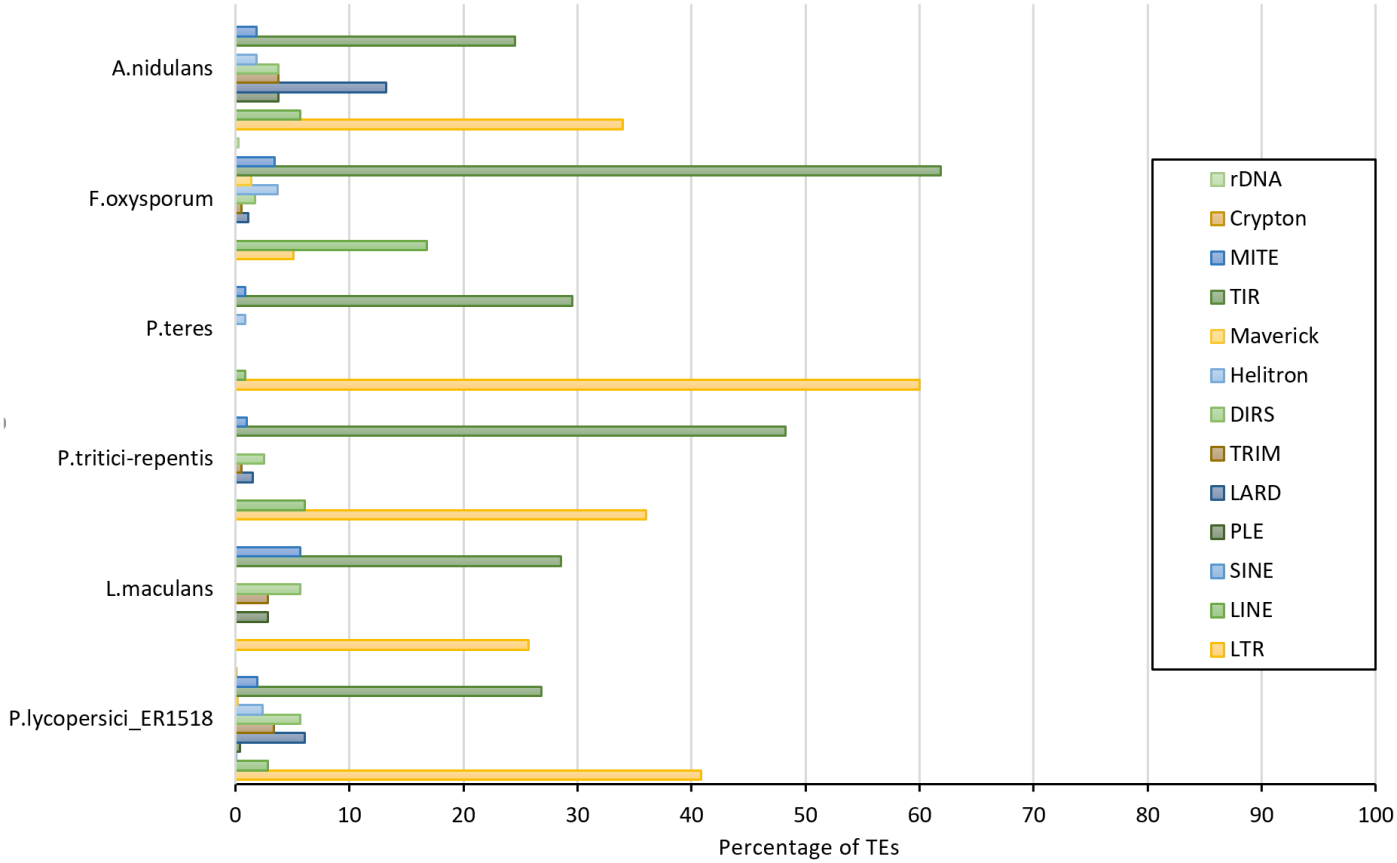
Repeat content comparative analysis among *P*. *lycopersici* ER1518 and other five ascomycetes. Histogram of percentages of different TE categories respect to the total annotated TEs reported for each species.

*De novo* TE prediction in *P*. *lycopersici* ER1518 identified 15 TE super families. In particular, *P*. *lycopersici* Class I TEs covered the 44.1% of the total repeat content while Class II TEs covered the 38.1%. With respect to each class, LTR retrotransposons (Class I) and TIR DNA transposons (Class II) accounted for the largest TE fraction, with a percentage of ~39.7% and ~32.8%, respectively (Fig 4 and S11 Table). Similar results were shown in other *Dothidiomycetes*, like *P*. *teres* and *P*. *tritici-repentis*, but not in *L*. *maculans*, which exhibited a remarkable expansion of Class I TEs, mainly LTR retrotransposons [44], whereas, *F*. *oxysporum* repeats were prevalently classified as Class II TEs [45], both in terms of percentages and copy numbers (S11 and S12 Tables). Among Class I elements, Copia and Gypsy were the most abundant in *P*. *lycopersici* ER1518 (Table 4), in agreement with the majority of fungal genomes.

A discrete fraction of *P*. *lycopersici* repeats consists of uncharacterized sequences (9,16%) which have no similarity to protein domains or structural features associated with known repeats. In addition, a discrete amount of non-autonomous TEs has emerged, including LARD and TRIM (Class I) and MITEs (Class II) families (Table 4 and S11 Table). These elements lack one or more genes for transposition but can be activated by the autonomous transposable elements. The presence of a large fraction of non-autonomous TEs in *P*. *lycopersici* suggests a high level of ectopic recombination between sequences of transposable elements. It is well documented the ability of TEs to move through the genome and to produce new phenotypes by creating new genes and re-modelling the existing ones [46–49]. The gene and TE annotation of *P*. *lycopersici* ER1518 isolate coupled with preliminary comparative analysis allowed to highlight interesting features relevant to the biological life traits of this pathogen. The possible association of putative protein-coding genes with TEs was investigated, based on the annotation proximity on the genomic sequence (Fig 3), obtaining that 42 of these predicted genes were functionally annotated as heterokaryon incompatibility protein-coding genes. As previously reported, the HET protein family is significantly expanded in *P*. *lycopersici*. Therefore, the proximity of *het* domains to repetitive elements (42 genes in 32 genomic unitigs) suggests the putative need for this fungal pathogen, where asexual reproduction is predominant, to increase the rate of evolution of these loci which contribute to genetic recombination.

Although *P*. *lycopersici* ER1518 possesses high repeat content like *L*. *maculans* (S10 Table), it has not the same distribution of AT-rich regions. In a recent work [39], the distribution of AT-rich regions was analysed in many fungal genomes, including *P*. *lycopersici* ER1211 and *L*. *maculans*. While, approximately, one third of *L*. *maculans* genome consists of AT-rich regions, only ~10% of *P*. *lycopersici* ER1211 genome consists of AT-rich regions (S2 Table of that study). Moreover, the high frequency of TpA dinucleotide in *P*. *lycopersici* ER1211 and other fungal genomes, reported in that work, is a strong indicator of RIP activity in these species. OcculterCut [39] was also used for analysing *P*. *lycopersici* ER1518 assembled genome and the results have been reported in S13 Table, together with those obtained for *P*. *lycopersici* ER1211, *N*. *crassa*, *L*. *maculans* and *A*. *brassicicola* by Testa *et al*. [39]. *P*. *lycopersici* ER1518 AT-rich component consists of 13% of total genome assembly, a value comparable to that obtained for *P*. *lycopersici* ER1211 and *N*. *crassa* (S13 Table). These results, together with the high frequency of TpA dinucleotide, already reported, confirm the presence of AT-rich regions and RIP activity in *P*. *lycopersici* species. In plant fungal pathogens, the interest in AT-rich regions has emerged by observations, of genes encoding effector-like proteins within or close to AT-rich regions [50]. From an evolution point of view, it has been proposed that pathogenic fungi with putative effector genes near AT-rich regions have the advantage to rapidly re-arrange these genes in response to new resistance genes developed by the host. OcculterCut analysis additionally reported the localization of 10 predicted genes annotated in AT-rich regions. Five of them are annotated as hypothetical proteins or have no hit in public databases, while the other five genes have a functional annotation in Pfam database: an antibiotic biosynthesis monooxygenase (Abm), two alpha/beta-hydrolases, an acetyltransferase (GNAT) and a short chain dehydrogenase/reductase (SDR). All the enzymes belonging to these families have a role in host-pathogen interaction, in a direct or indirect way. Abm converts endogenous free jasmonic acid into 12OH-JA which is secreted during host penetration in the model rice blast fungus *Magnaporthe oryzae*, and helps evading the defence response. Also *M*. *oryzae* members of the SDR family such as trihydroxynaphthalene reductase (3HNR) are key enzymes for fungal melanin biosynthesis, which is required for pathogenicity in this fungus. Catalytic members in the alpha/beta-hydrolase superfamily include acetylcholinesterase, carboxylesterase, lipase, cutinase, thioesterase, and other hydrolases; some of them containing predicted homologs from different fungal species, while some other existing as broad host-range pathogens. Finally, acetyltransferase activity is involved in histone acetylation and transcription signalling, very important for fungal pathogenesis.

### Identification of putative effectors

Many Dothideomycetes produce effectors to facilitate host infection [50]. The 14,186 *P*. *lycopersici* ER1518 predicted proteins were analysed to predict if they were likely to be secreted signal peptides and test if they were putative effectors. The analyses with Phobius [29] and EffectorP [30] resulted in 988 predicted signal peptides and 172 putative effectors genes, respectively. Among these putative effectors, 155 were functionally annotated in at least one of the databases examined (NCBI, Blast2GO, PFAM and dbCAN), while the remaining 17 may be considered putative unique effectors, because they were not annotated in any of the database taken in consideration. Among the putative effectors, about 13.5% were annotated in dbCAN, confirming the importance of some Carbohydrate-active enzyme families, expanded in both *P*. *lycopersici* sequenced isolates (Fig 1). Among these families, the AA9 family (formerly known as glycosyl hydrolase family 61 or GH61) accounts for 24% of dbCAN annotated putative effectors and includes lytic polysaccharide monooxygenases (LPMOs), able to cleave cellulose chains with oxidation of various carbons in a synergic activity with classical cellulases. Previously, an enzyme belonging to this family, named PlEGL1, has been isolated from *P*. *lycopersici* ER 1211 and functional characterized [51]. In the infected tomato plants the expression level of *Plegl1* was positively correlated to the development of the disease and this gene has been identified also among the putative effectors of *P*. *lycopersici* ER1518. This finding now strengthens the hypothesis of a putative effector role of this factor in the development of the necrotic lesions on infected roots, characteristic symptoms of Corky Root Rot disease caused by this fungal pathogen. The remaining fraction of estimated putative effectors included reductases, transcription factors, hydrophobins, membrane transporter families domains, as major facilitator superfamily (MFS), a family expanded in both *P*. *lycopersici* sequenced isolates. Globally, 59.3% of annotated genes showed homology to species belonging to Pleosporales order, which includes also *P*. *lycopersici*, in agreement with previously performed and present phylogenetic analyses (Fig 2, S3 and S4 Figs).

A major role in effector evolution in fungal plant pathogens is played by TEs [16,52,53]. In sight of this, the association of *P*. *lycopersici* ER1518 genes with transposable elements has been investigated. By analysing genes annotated in the 2 kb region downstream of the repeats, it was discovered a Zn2Cys6 binuclear cluster transcription factor homologous to the putative factor Pf2 identified in some important fungal pathogens belonging to Pleosporales order, as *Alternaria brassicicola* [19], *Parastagonospora nodorum* and *Pyrenophora tritici-repentis* [20]. In these pathogens *Pf2* regulates necrotrophic effector genes expression and host-specific virulence. Sequence similarity was detected between *A*. *brassicicola AbPf2* protein and a *P*. *lycopersici* ER1518 predicted protein (64.5% coverage and 77.6% identity). Hereafter, we refer to the *P*. *lycopersici* putative Pf2 gene homolog as *PlPf2*. A similar sequence identity was found with *Pf2* factors of other well-known Pleosporales pathogens, as *L*. *maculans*, *P*. *tritici-repentis* and *P*. *teres* (S5 Fig). Curiously, in pathogens with the highest TEs content, as *P*. *lycopersici*, *L*. *maculans* and *P*. *tritici-repentis*, *Pf2* gene is located close or between transposable elements, while in *A*. *brassicicola* and *P*. *teres*, which have a lower percentage of repeated sequences (9.7 and 3.4%, respectively), *Pf2* is not associated to transposable elements. Since this is the first report of a putative transcription factor in *P*. *lycopersici*, it will be interesting to investigate the role of *PlPf2* in regulating the expression of putative effector genes, which may contribute also to understand the evolutionary history of the Pf2 transcription factors, which until now seem exclusive only of Pleosporales [20]. Identification of these signals is fundamental for the knowledge of the pathogenic behaviour of one of the major soil-borne fungal pathogens of tomato.

## Conclusions

In this study, by using long-read-based SMRT sequencing technology, the quality of the genome assembly of the pathogenic fungus *P*. *lycopersici* has been improved. The availability of this new isolate’s gapless genome has enabled the in depth analysis of the gene content of *P*. *lycopersici* species and the identification of transposons and other repetitive sequences, which represent more than 30% of the total genome. These findings have given new insights into the biological complexity of this non-model pathogenic fungus, as the exceptional expansion of *het* gene family, linked to its mechanisms of reproduction, and the putative association of these proteins to repetitive sequences, possibly indicating mechanisms of recombination alternative to sexual reproduction. The completeness of *P*. *lycopersici* ER1518 genome sequence allowed also the identification of some putative effectors and a transcription factor with putative effector-related features relevant for virulence in plant pathogens. In the near future, the successful functional characterization of some of these putative effectors will be noteworthy, both for the understanding of *P*. *lycopersici* pathogenic behaviour and for the development of strategic methods for disease control.

## Supporting information

S1 Fig Visual comparison of sequence length distribution between *P*. *lycopersici* isolates. (TIF)

S2 Fig Alignment and coverage of *P*. *lycopersici* ER1518 and ER 1211. (TIF)

S3 Fig Phylogenetic tree of GH61 protein of *P*. *lycopersici* ER1518 and other ten ascomycetes obtained with PhyML 3.0 and drawn with MEGA7. (TIF)

S4 Fig Phylogenetic tree of HET2 protein of *P*. *lycopersici* ER1518 and other ten ascomycetes obtained with PhyML 3.0 and drawn with MEGA7. (TIF)

S5 Fig Multi-alignment of amino acid sequences of Pf2 transcription factor. (TIF)

S1 Table CEGMA analysis results for *P*. *lycopersici* ER1518. (XLSX)

S2 Table Assembly comparison between the two *P*. *lycopersici* isolates. (XLSX)

S3 Table Statistics of *P*. *lycopersici* ER1518 functional annotation. (XLSX)

S4 Table Heterokaryon incompatibility proteins related domains. (XLSX)

S5 Table Major membrane transporter families domains. (XLSX)

S6 Table CAZyme domains comparison. (XLSX)

S7 Table List of Carbohydrate-degrading enzymes in *P*. *lycopersici* and other fungi. (XLSX)

S8 Table OrthoMCL groups of *P*. *lycopersici* ER1518 and other 10 ascomycetes. (XLSX)

S9 Table Functional annotation of *P*. *lycopersici* ER1518 OrthoMCL singletons. (XLSX)

S10 Table Summary statistics of genome repetitive content analysis. (XLSX)

S11 Table Percentage of different TEs respect to the total annotated repeats in six fungal genomes. (XLSX)

S12 Table Copy numbers of different TEs respect to the total annotated repeats in six fungal genomes. (XLSX)

S13 Table AT-rich regions distribution in both *P*. *lycopersici* isolates and other ascomycetes. (XLSX)
