# Supplementary figures and images for: The genome assembly of the fungal pathogen Pyrenochaeta lycopersici from Single-Molecule Real-Time sequencing sheds new light on its biological complexity
Source: PLoS One. 2018 Jul 6;13(7):e0200217. doi: 10.1371/journal.pone.0200217 (PMC6034849; doi:10.1371/journal.pone.0200217)

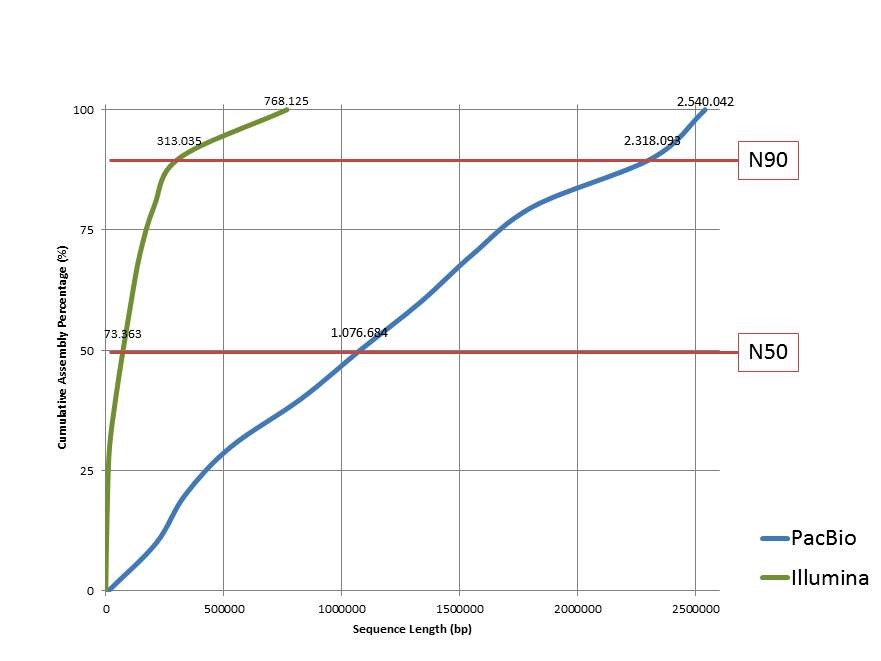

Supplement: S1 Fig — (TIF) [file pone.0200217.s001.tif]

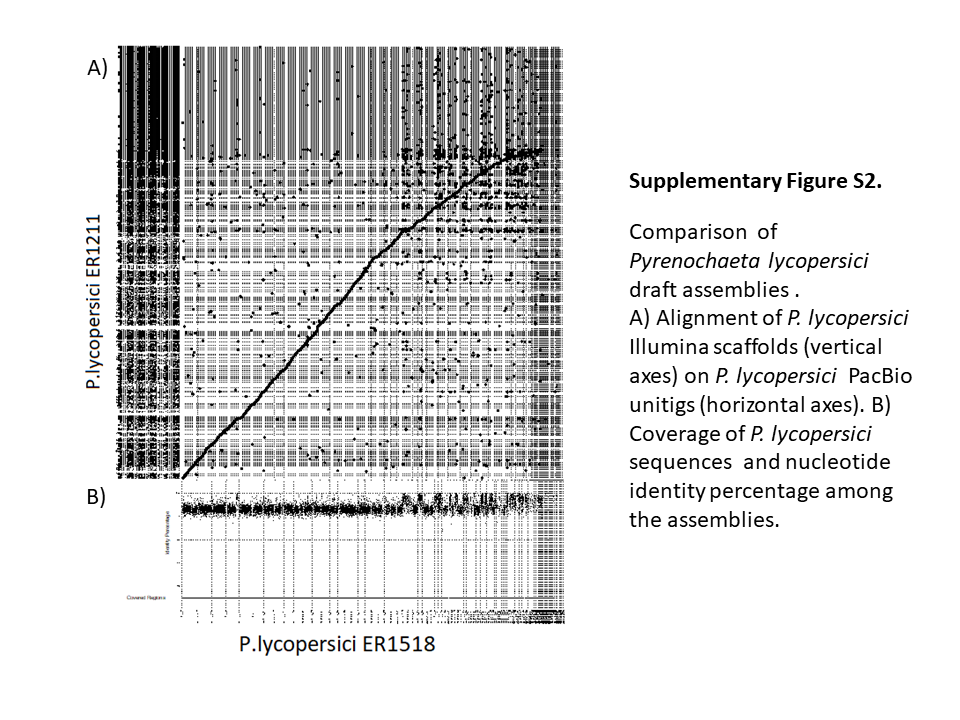

Supplement: S2 Fig — (TIF) [file pone.0200217.s002.tif]

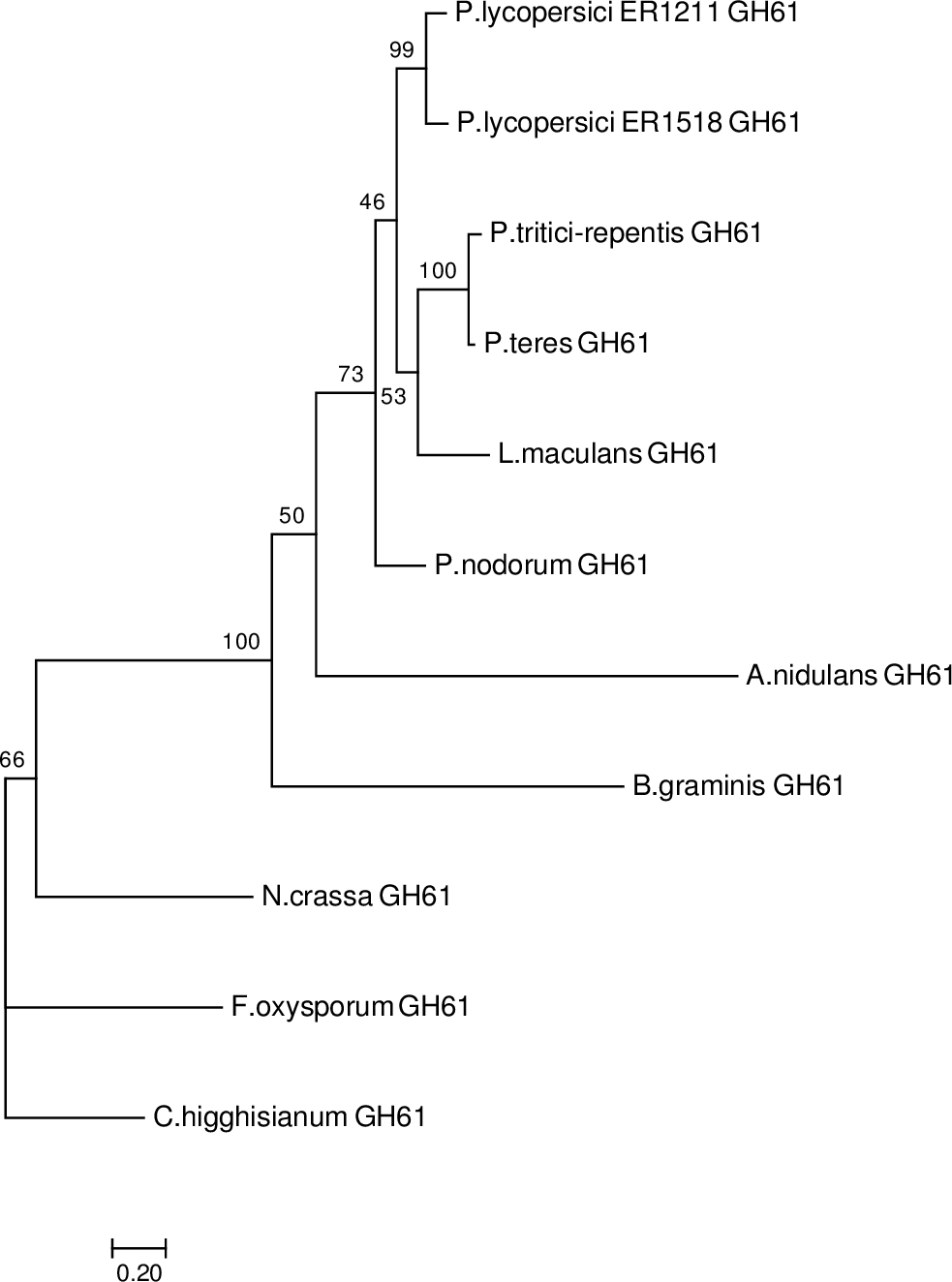

Supplement: S3 Fig — (TIF) [file pone.0200217.s003.tif]

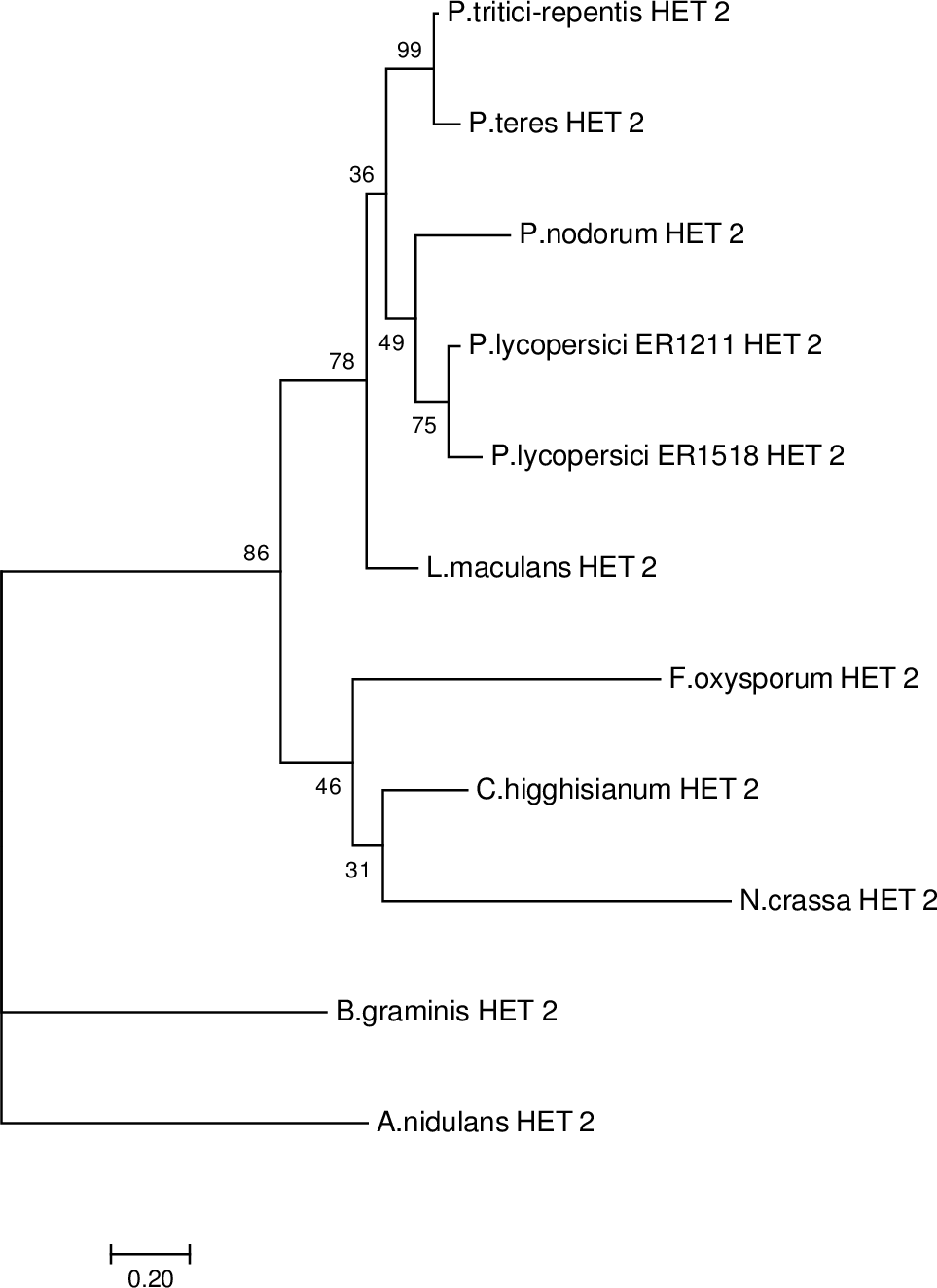

Supplement: S4 Fig — (TIF) [file pone.0200217.s004.tif]

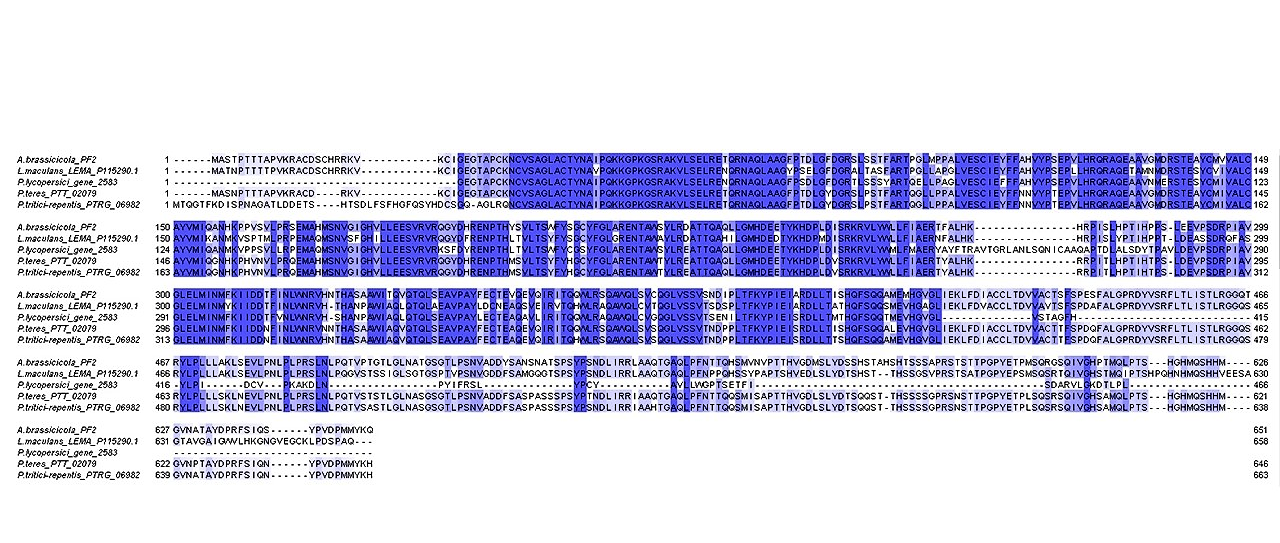

Supplement: S5 Fig — (TIF) [file pone.0200217.s005.tif]
